# Developing and implementing a model of equitable distribution of mentorship in districts with spatial inequities and maldistribution of human resources for maternal and newborn care in Rwanda

**DOI:** 10.1186/s12913-021-06764-y

**Published:** 2021-07-27

**Authors:** Anaclet Ngabonzima, Cynthia Kenyon, Daniel Kpienbaareh, Isaac Luginaah, Gisele Mukunde, Celestin Hategeka, David F. Cechetto

**Affiliations:** 1 Economic Community for Central African States (ECCAS), Libreville, Gabon; 2 Neonatal - Perinatal Medicine, Schulich School of Medicine and Dentistry, University of Western Ontario, London, Canada; 3 Department of Geography and Environment, University of Western Ontario, Ontario N6A 5C1 London, Canada; 4 Department of Anatomy & Cell Biology, Schulich School of Medicine & Dentistry, University of Western Ontario, N6A 5C1 London, Ontario Canada; 5 Centre for Health Services and Policy Research, School of Population and Public Health, Faculty of Medicine, University of British Columbia, BC Vancouver, Canada

**Keywords:** Onsite mentorship, Maternal health, Perinatal outcomes, Rwanda

## Abstract

**Background:**

The shortage of health care providers (HCPs) and inequity in their distribution along with the lack of sufficient and equal professional development opportunities in low-income countries contribute to the high mortality and morbidity of women and newborns. Strengthening skills and building the capacity of all HCPs involved in Maternal and Newborn Health (MNH) is essential to ensuring that mothers and newborns receive the required care in the period around birth. The Training, Support, and Access Model (TSAM) project identified onsite mentorship at primary care Health Centers (HCs) as an approach that could help reduce mortality and morbidity through capacity building of HCPs in Rwanda. This paper presents the results and lessons learnt through the design and implementation of a mentorship model and highlights some implications for future research.

**Methods:**

The design phase started with an assessment of the status of training in HCs to inform the selection of Hospital-Based Mentors (HBMs). These HBMs took different courses to become mentors. A clear process was established for engaging all stakeholders and to ensure ownership of the model. Then the HBMs conducted monthly visits to all 68 TSAM assigned HCs for 18 months and were extended later in 43 HCs of South. Upon completion of 6 visits, mentees were requested to assist their peers who are not participating in the mentoring programme through a process of peer mentoring to ensure sustainability after the project ends.

**Results:**

The onsite mentorship in HCs by the HBMs led to equal training of HCPs across all HCs regardless of the location of the HC. Research on this mentorship showed that the training improved the knowledge and self-efficacy of HCPs in managing postpartum haemorrhage (PPH) and newborn resuscitation. The lessons learned include that well trained midwives can conduct successful mentorships at lower levels in the healthcare system. The key challenge was the inconsistency of mentees due to a shortage of HCPs at the HC level.

**Conclusions:**

The initiation of onsite mentorship in HCs by HBMs with the support of the district health leaders resulted in consistent and equal mentoring at all HCs including those located in remote areas.

## Introduction

Globally, maternal mortality remains unacceptably high. In 2017, 295 000 women died during or following pregnancy and childbirth (approximately 810/day). The vast majority of these deaths occurred in low-resource settings and most could have been prevented [1]. Further, approximately 2.5 million newborns died in 2018, most of which were in Low-and Middle-Income Countries (LMICs) where newborns are nine times more likely to die in the first month of life compared to babies born in high-income countries [2]. Preterm birth, intrapartum-related complications, infections, and birth defects are the leading cause of neonatal deaths in LMICs [2, 3]. About the same number of babies in the LMICs are stillborn, with a stillbirth rate of over 18.4 per 1000 births [1].

A significant number of maternal and newborn related deaths in LMICs are linked to the shortage of qualified Health Care Providers (HCPs) who are needed to provide quality prenatal care, skilled birth attendance and emergency obstetric services - interventions crucial to reducing maternal and perinatal deaths [4, 5]. This shortage is complicated by a maldistribution of staff, inadequate training, a lack of professional development and equal opportunities, skills mix imbalance, high patient-doctor ratio, and increasingly complicated medical programmes and limited physical resources [6]. The lack of HCPs is a serious impediment to the provision of effective maternal and perinatal care in LMICs [5, 7]. In addition, gaps frequently exist in the implementation of effective Continuing Professional Development (CPD) strategies. As such, the limited staffs available are unable to build their competencies in the clinical domain, as well as develop skills in management, team building, professionalism, interpersonal communication, teaching and accountability [8–10].

Several studies have revealed that didactic training does not effectively translate knowledge into practice to address system-level barriers [11, 12]. However, mentorship programmes have been shown to improve the quality of care in many countries [13–15]. Consequently, the provision of CPD programmes like mentorship has been identified as necessary, especially in contexts dealing with HCPs limitations. Thus, there is a critical need to identify and invest in effective models to train and support HCPs [16, 17].

Rwanda has made great strides in the area of women’s and children’s health over recent decades including the attainment of Millennium Development Goals (MDGs) 4 and 5 [18–20]. Despite these achievements, the number of maternal deaths, under-five mortality, and stillbirths is still high [17, 18] and the leading causes of neonatal and maternal mortality are still preventable if skilled HCPs perform effective health measures during the perinatal period and in a continuum of care. This is the care that is provided in a seamless continuum that spans the home, the community, the health center and the hospital.

To address high morbidity and mortality rates likely due to insufficient competent HCPs in the health facilities, the TSAM project designed and implemented mentorship programmes in HCs as a way of strengthening the continuum of care while also building the capacity of HCPs to be able to deliver high-quality care during the perinatal period. TSAM for MNCH is a project with funding provided to the University of Western Ontario (Western) by Global Affairs Canada (GAC). TSAM’s mission is to improve Maternal, Newborn and Child Health (MNCH) in Rwanda by working with local partners to improve health service access and delivery.

Although there is the need to develop and implement effective models to train HCPs, there were no broad-based models for mentoring in the health centers in Rwanda. Furthermore, we believe that the model we developed based on making use of the midwives from District Hospitals (DHs) that were given extensive mentoring by an interprofessional group of experts from tertiary hospitals, to provide mentoring at the health centers in their hospital catchment area has not been used elsewhere in resource-poor countries [15].

## Methods

### Aim

 This paper aims to describe the development, implementation as well as results of the mentorship model for HCPs providing maternal and neonatal care in 68 health centers of the 3 districts of the Northern Province of Rwanda. The indicators that were assessed for the results included the distribution and consistency of mentoring to the catchment areas of all of the DHs included in this project. The data on the initial assessment of the study locations were also analysed.

### Design and settings

The 68 HCs involved in this study were located in Rulindo, Gicumbi and Gakenke districts in the Northern Province of Rwanda. The onsite mentorship programme was implemented by TSAM project and its stakeholders in the HCs in the three districts. These 3 districts were assigned to the TSAM project as per the Memorandum of Understanding between the project and the Ministry of Health. This research describes the mentorship programme. The design phase of this programme started with an initial assessment of the status of training in HCs in these districts. The results of this assessment informed the selection of midwives to serve as Hospital-Based Mentors (HBMs) for health care providers at the HCs. The research further describes the other steps of the design phase, namely the refresher courses on Emergency, Obstetric Neonatal Care (EmONC) for selected Hospital Based Mentors and training on the mentoring approach to be adopted. These HBMs also benefited from other courses with cross-cutting themes such as ethics, inter-professional collaboration, gender, maternal mental health and Gender-Based Violence (GBV). The initial training makes this mentorship programme unique, as the additional training was designed to allow HBMs to manage the mothers and newborns in an integrated manner. In addition, HBMs were drawn from the mentees who had followed the mentoring programme at the respective hospitals under the same project framework. Upgrading of some mentees to HBMs through cascades of special training presents the second unique aspect of our mentorship programme. The fact that mentors were drawn from a cadre of DH mentees allowed them to conduct mentorship more effectively.

The design phase involved information meetings to establish a clear process for engaging all stakeholders and to ensure ownership of the model. Following the initial assessment and training, 23 HBMs conducted monthly visits to the 68 HCs located in catchment areas of five TSAM assigned hospitals in North for 18 months, from October 2018 to March 2020. This model was extended later in other HCs located in the catchment area of 5 hospitals in the Southern Province. Participants to the mentoring programme described in this manuscript were nurses or midwives providing maternal and/or neonatal care in 68 HCs of 3 concerned districts in the Northern province.

### Data collection and analysis

Data on the number of mentees who attended the mentorship programme during its lifetime as well as the data on the initial assessment were analysed for this study. The data were entered in a database designed for the project using Excel. Analysis was done in Microsoft Excel to generate tables and descriptive statistics. Locations of HCs were collected using Global Positioning System (GPS) devices and the coordinates were used to generate maps in ArcGIS 10.7. The geographic data were analysed to identify spatial disparity of midwives using data on the number of midwives and population density for each hospital catchment area. Population data were obtained from the Health Information System of the hospital. In addition, data were analysed according to the number of nurses and midwives in the health centers that had received training on EmONC. Mentoring data was collected on the number of mentoring visits in the DH catchment areas segregated by gender for the nurse and midwife mentees. Also, data on the number of mentoring visits were collected based on the professional qualification of the midwives and nurses.

### Outcomes of the need assessment

Prior to designing the mentoring programme at the HC level, a rapid assessment in health facilities located in TSAM-assigned hospitals was conducted by researchers working on the project. The assessment aimed to determine the availability of staff who provide MNH care and the status of training on MNH for those staff. The results of the assessment allowed the project and its stakeholders not only to know the number of mentees that would be available but also it informed the initial selection of competent hospital-based mentors (HBMs). This assessment was conducted cognizant of the fact that over the past 2 decades, HCPs have received some off-site training related to maternal and newborn care [14, 19, 21]. The findings of the assessment are presented below:

### Availability of midwives

**Fig. 1:**
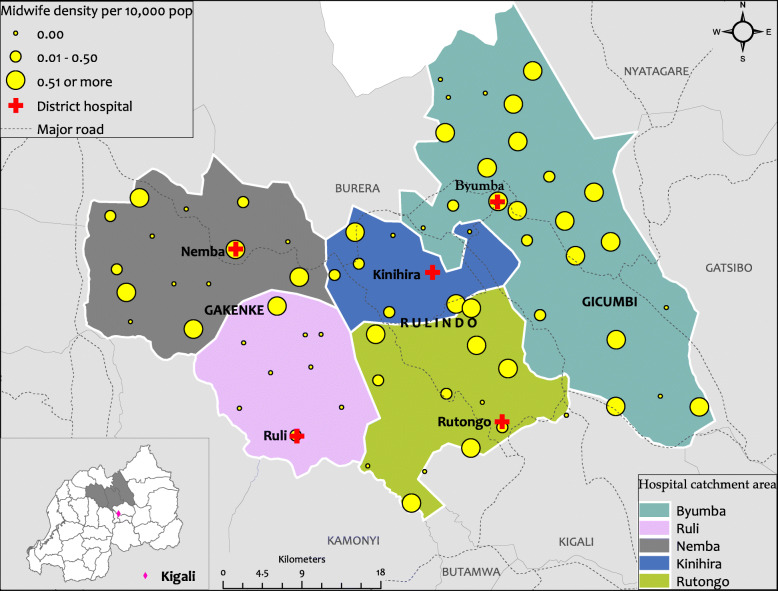
Spatial distribution of the midwives per HCs in Rulindo, Gicumbi and Gakenke districts. This Figure shows the spatial distribution of the number of midwives per HCs in Rulindo, Gicumbi and Gakenke districts. Produced using ESRI 2019. ArcGIS Desktop: Release 10.7.1. Redlands, CA: Environmental Systems

The results revealed that efforts are being made to equip maternity departments of HCs with midwives. Of the 68 HCs located in the catchment area of TSAM-assigned hospitals, 43 (63 %) had at least one midwife by the time of the assessment. However, as seen in Fig. 1 below, the density of midwives per 10000 population varied widely both within and across the districts as well as across the hospital catchment areas. HCs that were easily accessible had more midwives than those in more remote areas. For example, it is easy to realize that the density of midwives in Gicumbi district is higher compared to Gakenke district while even in Gakenke district itself, the density of midwives is higher in the catchment area of Nemba hospital than that of Ruli hospital. This disparity points to the need for the development of the TSAM mentorship programme to build the capacity of HCPs providing MNH in all HCs.

### Spatial disparity of midwives and nurses trained on Emergency Obstetric and Newborn Care (EmONC)

**Fig. 2:**
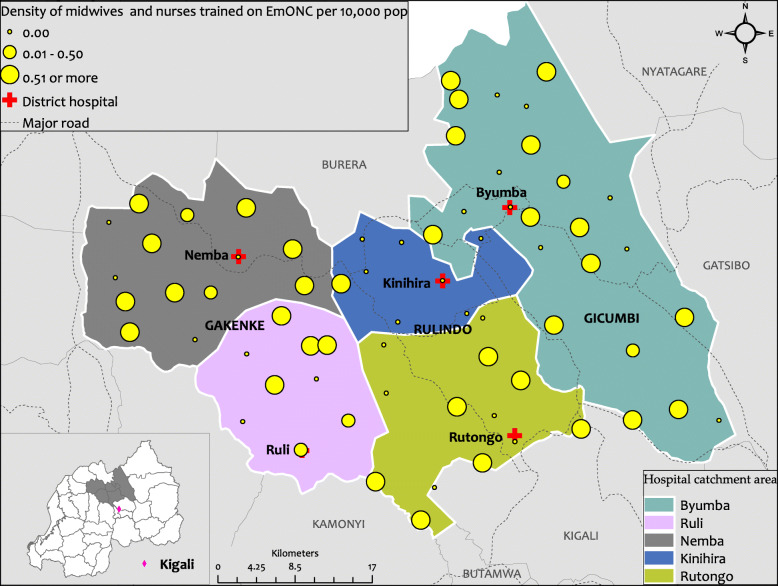
Spatial distribution of midwives trained on EmONC per HCs in Rulindo, Gicumbi and Gakenke districts. This Figure represents the spatial distribution of midwives who benefited from the training on EmONC in Rulindo, Gicumbi and Gakenke districts. Produced using ESRI 2019. ArcGIS Desktop: Release 10.7.1. Redlands, CA: Environmental Systems

Apart from the availability of midwives in the HCs, the assessment revealed that there is an inequity in terms of densities of health care providers who benefited from the training on the EmONC across hospital catchment areas and districts. As shown on Fig. 2 below, the density of health care providers who received training on EmONC per 10000 population is higher in Gicumbi district and Gakenke district than in Rulindo district. However, even in the Gakenke district, the density is higher in the catchment area of Nemba hospital than in Ruli hospital catchment area. Likewise, even in Rulindo district which has a poor density, there is a disparity in the catchment area between Kinihira and Rutongo, further highlighting the need for the TSAM programme to implement a mentorship programme that focuses on EmONC to reach all HCs to provide consistent mentoring despite the disparities.

### Spatial distribution of Health Care Providers (HCPs) trained in Essential Newborn Care (ENC)

**Fig. 3:**
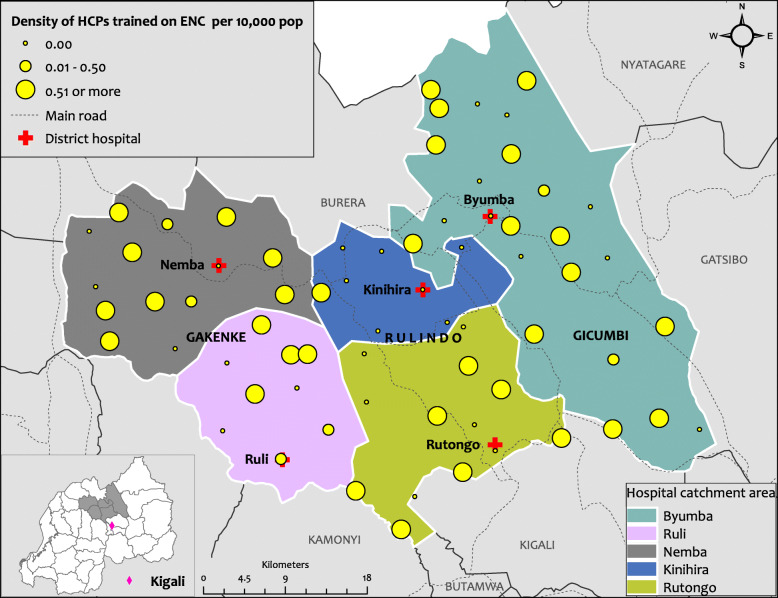
Spatial distribution of HCP trained on ENC per HC in Rulindo, Gicumbi and Gakenke districts. This Figure shows the spatial distribution of health care providers who benefited from the training on ENC in Rulindo, Gicumbi and Gakenke districts. Produced using ESRI 2019. ArcGIS Desktop: Release 10.7.1. Redlands, CA: Environmental Systems

The initial assessment also allowed the project and its stakeholders to examine the spatial distribution of HCPs with additional training on Essential Newborn Care (ENC). As with the EmONC, there were geographical disparities with HCPs trained in ENC which is thought to be helpful for staff providing care to mothers and newborns (Fig. 3).

### Rationale of the TSAM mentorship model

Mentorship is a flexible teaching and learning process that serves specific objectives of the HCPs and health care services [13–15, 21]. Given the disparities in maternal health services [22] and previous training programs that were provided to midwives, the TSAM project targeted staff providing care to women and newborns. This approach can reach many people at the same time without disturbing the routine work of service providers. More importantly, some maternity departments of HCs are staffed with midwives who have only recently graduated. Apart from the fact that they have not received any additional training to strengthen their capacity in the area of EmONC, they have limited opportunities for CPD beyond pre-service training and have limited access to experienced clinicians from whom they could learn key skills. For these HCPs, pre-service training and refresher courses alone may not translate into improved maternal health service delivery. Hence, the TSAM mentorship programme aimed to provide the bridge between traditional didactic training to hands-on practical training approaches. This was based on the underlying assumption that skills acquired during pre-service training are usually lost in the absence of CPD. Through TSAM’s mentorship approach, key essential MNH skills were imparted to enable nurses and midwives to effectively perform tasks that they did not feel confident in performing, either due to lack of knowledge, hands-on skills or both. The following section describes the development, implementation, and preliminary outputs of an onsite mentorship programme in the TSAM-assigned HCs in Rwanda.

### Mentorship model development

The mentorship model was implemented by HBMs who were midwives practicing in the DHs responsible for oversight of the HCs. The majority of these midwives had been mentored in a separate programme for HCPs at the DH level by national mentors with the support of the same project [22]. Potential mentors were selected with that input from the administration of DHs and the selection considered the following key criteria as per the mentorship guidelines of the Ministry of Health [23].

The mentorship model development consisted of several phases. The first was providing a refresher course on Emergency Obstetric and Newborn Care (EmONC) for 25 potential mentors. The candidate HBMs was proportional to the number of HCs within the catchment area of each hospital. The second phase consisted of providing training in mentoring and Cross-Cutting Themes (CCTs) including Gender, Ethics and Inter-Professional Collaboration to successful candidates to EmONC refresher training. Thirdly, there were induction meetings held for each DH to introduce the programme and ensure it is owned by beneficiaries. The final phases were the implementation of the onsite mentorship visits followed by monitoring and evaluation activities. Once again, it is worthy to mention that HBMs were drawn from former mentees.

### Onsite mentorship field visits and related coordination

After a successful design phase, each HBM was assigned to between 2 and 3 HCs. The mentorship field visits were organized for all 68 HCs in the Northern Province. One-day monthly visits were conducted by HBMs from October 2018 to March 2020 (18 months). During the mentoring period, key services areas mentored include the labor ward, post-natal care and antenatal care services. Activities carried out by HBMs include management of cases with the mentees, bedside teaching, presentation on key selected topics in morning staff meetings based on the needs, and training of mentees using simulation. Logbooks were used to track the participation of mentees in different mentorship activities. The topics covered by HBMs were the components of Essential Newborn Care (ENC) and those of EmONC. HBMs had benefited from refresher courses on these topics to be covered. During the mentorship period, different activities were completed by mentors and mentees including ward round, assisted delivery, newborn resuscitation, and post natal care. In addition, mannequins were used to teach skills for different components. Upon completion of each of the visits, reports were written and submitted to the director of nursing at each hospital for compiling the report at the hospital level.

### Coordination of the mentorship activities

The overall coordination of the mentoring programme was done by a CPD programme manager within the TSAM project. The formats of the reports by the mentors were developed by the TSAM project CPD team and presented during both the training on mentoring and induction meetings. The completed reports were then submitted to the CPD manager for the TSAM project to be compiled and analyzed and a report prepared.

### Mentorship monitoring and evaluation meetings

To ensure that the mentorship model was implemented as designed and experiences were shared, bi-annual evaluation meetings were organized for each hospital. These meetings brought together the same participants as those who attended the induction meetings before initiating the mentorship in HCs. The monitoring meetings aimed to share the key messages from the report of the mentorship visits, discuss the successes and challenges of the mentorship visits as well as develop the strategies to overcome the challenges. The key points that emerged from the coordination meetings are summarized below:

- The mentoring programme is helping to significantly improve the quality of care in health centers, this was mentioned based on the way HCs appreciated the improvement of the knowledge and skills of HCPs.
- The mentoring programme is conducted the way it was designed.
- The mentoring programme is different from any other form of supervision at the HC. This is because mentees realized that mentors are not supervising what they are doing but that they are working together while providing feedback. They were used to supervisors where the later come to evaluate only what they did.
- Mannequins were distributed to all HCs to enhance the knowledge co-sharing by the mentorship programme using simulation to achieve its goals.
- There is a need to strengthen the consistency of mentees during the mentorship visits. The fact that some mentees have not been consistent due to the shortage of HCPs was mentioned here as one of the key challenges.

## Results

From October 2018 to March 2020 (18 months), each of the 68 HCs located in the catchment area of 5 hospitals in Rulindo, Gicumbi and Gakenke benefited from mentorship monthly visits (Table 1). A total of 196 HCPs benefited from the mentorship visits for an average of about 3 HCPs for each HC. Given the structure of the HCs where most of them have one or two HCPs providing MNCH care, it was planned to mentor at least two HCPs per HC to ensure the consistency of mentees. In terms of density of mentees per population, the results showed that we managed to reach at least one health care provider per 10,000 population for the catchment areas of the five hospitals. The density of each health care provider per 10,000 population varied between 1 and 2 with an average of 1.3, suggesting mentorship was done equitably. We encouraged that once a mentee attended at least 6 visits, s/he starts mentoring her/his colleagues (peer mentoring) who have not been able to be part of the programme to ensure the knowledge and skills reach all staff of the HC. Among all 196 mentees, 146 (74.5 %) of them benefited from at least six or more mentorship visits. The mentees were nurses or midwives providing MNH care in the HCs. Of 146 mentees who completed at least six visits, 33 (22.6 %) were males while 113 were female (77.3 %). Of the 68 HCs, 58 (85 %) had at least two HCPs who participated in at least 6 visits and 15 HCs among them had HCPs who attended more than two visits. Of the remaining 10 HCs, all had one HCP participating in at least six visits. Once again, these results suggest that the mentoring programme by HBMs succeeded in mentoring the HCPs providing care to mothers and newborns in all HCs in a relatively equitable manner. Also, most of the staff benefited from presentations of mentors conducted during the morning staff meetings on specific key topics directed toward quality improvement.

**Table 1:** Mentees (HCPs) and mentorship visits per gender after 18 monthly visits

Hospital	Byumba		Kinihira		Nemba		Ruli		Rutongo		Total
No of visits/Sex	M	F	M	F	M	F	M	F	M	F	
More than 12	5	25	3	8	6	10	-	9	3	10	79
From 6 to 12 visits	8	11	2	6	1	9	2	11	3	14	67
Total per sex	13	36	5	14	7	19	2	20	6	24	146
Total per hospital	49		19		26		22		30		146
Number of HC	24		8		14		9		13		68
Average per HC	2		2.4		2		2.5		2.3		2.1
Number of served population in the hospital catchment area	438 563		145249		237274		111 656		192 092		1124834
Average mentees per 10000 population	1.1		1.3		1.1		1.9		1.5		1.3

*M *Male, *F *Female

As per Table 2 below, out of 146 mentees (HCPs) who completed at least six visits, 49 (33.6 %) are midwives, 60 (41.1 %) are A1 nurses while 37 (25.3 %) are nurses A2.

**Table 2:** Mentees per hospital and per qualification

Hospital/Qualification	Byumba	Kinihira	Nemba	Ruli	Rutongo	Total
Nurses A2	7	8	7	5	10	37 (25.3 %)
Nurses A1	21	4	14	11	10	60 (41.1 %)
Midwives	21	7	5	6	10	49 (33.6 %)
Total	49	19	26	22	30	146 (100 %)

A1 = nurses with a 3-year diploma; A2 = nurses with secondary school training

The data in Tables 1 and 2 indicate two things. First, the distribution of mentoring visits is based on a gender-segregated count. Since the mentees were primarily A1 and A2 nurses and midwives, it is not expected that the number of males and females being mentored would be equal. Furthermore, it shows that even though the total number of mentoring visits was not the same for each hospital catchment area, the average number of visits for each health center is relatively equal due to the differences in the number of health centers for each hospital. Finally, it illustrates that mentees from 3 types of professional qualifications benefitted from the mentoring.

**Fig. 4:**
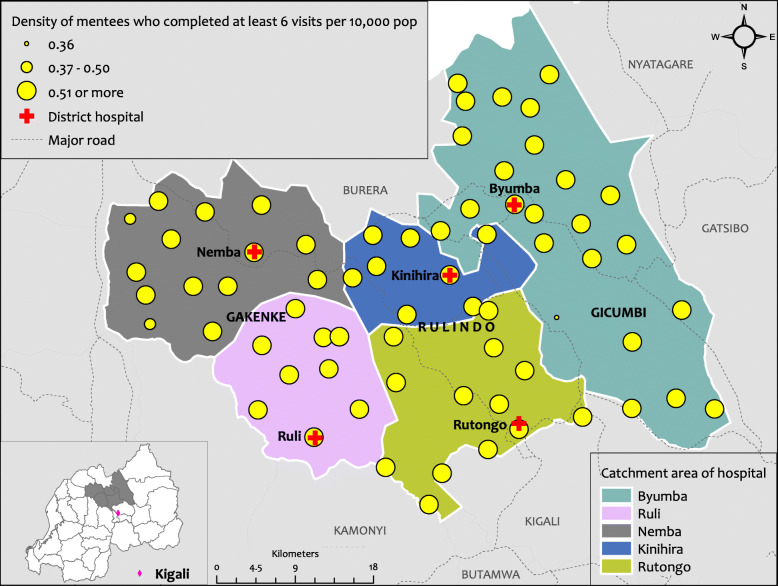
Spatial distribution of HCPs who benefited from at least six mentorship visits. This Figure shows the spatial distribution of health care providers who benefited from at least six mentorship visits per HC in Rulindo, Gicumbi and Gakenke districts. Produced using ESRI 2019. ArcGIS Desktop: Release 10.7.1. Redlands, CA: Environmental Systems

Additional analysis examined the spatial distribution of mentees in the HCS (Fig. 4). The map indicates that there is a relatively even distribution of mentoring throughout all targeted districts and no HC failed to have at least one HCP who attended at least 6 visits. A minimum of 6 visits is the number of visits required to be validated as a HCP who has completed the mentorship programme.

### Completed and ongoing research on this mentorship model

The findings of studies, arising from the TSAM program, published in peer-reviewed journals which evaluated the mentorship programme revealed that the mentorship considerably improved the knowledge and self-efficacy of HCPs in managing PPH and newborn resuscitation in Rwanda [24, 25]. This is very encouraging in the context of countries with high maternal mortality and morbidity like Rwanda and such kind of a mentoring programme in an equitable manner may contribute to the attainment of SDGs related to maternal and newborn health.

### Challenges

Despite the demonstrated benefits of the mentorship programme, some challenges were identified through regular mentorship supervision and even through the mentor’s report. These must be overcomed if it is to be integrated into the mainstream health system of the country. They include the limited number of HCPs in HCs and more importantly in hospitals where the HBMs are working which does not allow mentoring a big number of mentees at the same time, lack of some essential equipment at HC and the staff turnover. Particularly for hospitals where mentors are working, it required to rearrange the schedules to ensure that mentorship was integrated into the monthly schedules, but a high commitment of the hospitals was required to succeed in this.

## Discussion

The TSAM project mentorship model was designed and implemented to provide training and support for health care providers in health centers to help improve maternal and newborn health in Rwanda. The goal of the project was to use mentoring to improve specialized care for mothers, newborns and children at all levels of health care. This paper describes the results of the mentorship programme after 18 months of implementation in 68 health centers located in the catchment areas of five hospitals in the Northern Province of Rwanda. The model was developed after conducting an assessment to determine the status of trained staff in MNC in the health centers. The assessment showed an inequity and maldistribution of HCPs who had previously benefited from the classic modular training in MNH off-site from the HCs and hospitals. The mentorship was then provided at all the TSAM-assigned health centers to improve the skills of the HCPs in those health facilities hoping to reduce maternal and newborn mortality.

Analysis of the results of the programme indicated that the mentorship training programme led to equity in the capacity of HCPs regardless of the location of the health center. One hundred and forty-six (146) HCPs took part in 6 or more mentorship visits, translating into an average of 2 mentees who completed at least six visits per HC. Looking at the consistency of mentoring and taking into account the discussion with all involved people during the coordination meetings, mentorship of HCPs has potentially improved their skills and performance at all levels of the health system. This is consistent with the findings of studies that evaluated this mentorship programme and which revealed that the mentorship improved the knowledge and self-efficacy of health care providers in managing PPH and newborn resuscitation in Rwanda [24, 25]. According to the findings of the research to evaluate this mentorship model, knowledge increased from 68 % before mentorship to 87 % and self-efficacy from 6.9 to 9.5 average score out of 10. In addition, results revealed that knowledge and self-efficacy correlated moderately positive at pre-mentorship (r = 0.214) and strongly positive at post-mentorship (*r* = 0.58) [24]. These findings are also consistent with the findings of other studies that have assessed mentorship programmes at both the facility and educational level and found that such mentorships are beneficial to the health providers and improve the quality of care delivery [13–15, 21].

The current successes of the TSAM project’s onsite mentorship model demonstrate the practicability of rapidly implementing a provider-centered mentorship programme that will deal with the quality of care and equity at both the individual and structural levels at the district level. Mentorship training, thus, has the potential to enhance the skill level of health care workers and improve the quality of care provided in rural Rwanda. In India, Jayanna et al. [26] found that an onsite mentoring programme improved the preparedness of the health care workers and the readiness of the health facility to address institutional births and related complications. Our findings are consistent with this observation.

Onsite mentorship programmes can ensure equity in access to skilled service providers and quality health care because it offers training tailored towards the trainee’s work situation. Thus, mentorship differs from in-class didactic training. This means that HCPs are trained where they are and in what they do, thus improving quality and equity in the number of skilled people in rural areas. In Rwanda, where, despite the achievement of the MDGs on maternal health care, disparities in the use of health services and quality of care still exist [22, 27]), such mentorship programmes can prove to be very useful in further improving access to skilled caregivers and improving equity. Angelini, [28] and Harrington, [29] as well as other authors, argue that mentorship provides career development opportunities for health workers and promotes a positive work environment where career satisfaction is guaranteed, leading to improved delivery and equity in access to providers due to retention of the skilled workers. In the case of the TSAM mentorship model, mentors from the DHs visited the various health centers to provide the appropriate training on EmONC and ENC to improve maternal and newborn health in the beneficiary health centers. Furthermore, the fact that the TSAM project trained local mentors who are midwives practicing in the DHs to conduct mentorship in the health centers of the catchment area of their respective hospitals may have improved assimilation of the training provided, resulting in the improvement in the quality of care. Using ‘foreign’ mentors from outside of the district may not achieve desirable results because they are less knowledgeable of local conditions, language, or health sector policies.

An additional component of a successful mentoring programme at the HCs is coordination at the district level. The TSAM programme managed this by having the mentor complete reports and after compilation and analysis, the report was shared with the hospital management team for action in case any issue was identified. It is part of supporting the DHs and their mandate to support the HCs in the mentoring programme. This is direct feedback to the management team at the DH and it supported the project efforts to be collaborative by making certain that the DH management team is aware of the experiences of the mentors. Another important factor is the involvement of Directors of Nursing in the supervision of the mentoring programme.

 Given the benefit of the TSAM onsite mentoring programme and similar ones such as the Mentoring and Enhanced Supervision at Health Centers (MESH) and their potential to further reduce maternal and neonatal deaths in Rwanda during the era of the Sustainable Development Goals (SDGs), there is a need to mainstream and regularize this model into the health care system. Mainstreaming this onsite model into the health delivery systems is especially useful in resource-poor settings where there are shortages of specialized health workers. Not only will this ensure that health care workers are up to date with the current trends in their fields, but it will also increase job satisfaction and lead to better performance and retention of skilled workers in rural areas, cognizant that job satisfaction is an important determinant of job retention [30]. It is worth noting that institutionalizing mentorship within HCs and hospitals will speed up their journey towards further reducing maternal and child deaths by 2030 [31].

Despite the demonstrated benefits of the mentorship programme, some challenges must be overcomed if the programme is to be effectively integrated into the mainstream health system of the country. These challenges include the limited number of HCPs in health centers and more importantly in hospitals where the mentors are working which does not allow mentoring a big number of mentees at the same time, lack of some essential equipment at health centers and the staff turnover [31]. Meanwhile, the mentorship can work in response to the limited number of health care providers in health facilities in contrast to the classic training as this takes place within the health facilities while the classic training requires the staff to leave the workplace for a certain period, thus disrupting the existing busy schedule of the facility. Despite the challenges, the mentorship programme is generally effective for several reasons. The programme allows for continuous onsite training by facilitating peer mentoring whereby the mentee progressively mentors their peers while at work. As result, the cost of improving the skills and self-efficacy is lower compared to off-site mentoring programmes and ultimately hospitals can integrate the mentorship activities in the regular schedules of the midwives to empower newly trained HCPs.

## Conclusions

In summary, after a year and a half of implementing the onsite mentorship in health centers, there has been equity in the number of skilled personnel in EmONC and ENC in the Northern Province of Rwanda. All health centers were reached monthly and disparities and inequities in terms of capacity building were avoided. We conclude that the programme generated a good number of mentees who have been consistent and trained by HBMs who were drawn from the former mentees with the hope that this will improve significantly the quality of care to women and newborns in Gakenke, Gicumbi and Rulindo districts. However, very close monitoring and coordination of the mentoring including regular supervision and feedback as well as the engagement of leaders at the facility level are key to success. The strategy to strengthen knowledge retention is required for the sustainability of the gains.

### Implication for future research

Researchers in the field of CPD for HCPs in primary health care facilities should consider researching job retention of mentees after some period of mentoring. The cost-effectiveness of the onsite mentorship should also be considered to compare the classic training and the onsite mentorship. Finally, researchers should examine whether onsite mentorship enhances job satisfaction and job retention among HCPs.

## Data Availability

The datasets used in this study may be available from the corresponding author upon a reasonable request.

## Abbreviations

CCTs: Cross Cutting Themes
CPD: Continuous Professional Development
DH: District Hospital
EmONC: Emergency, Obstetric Newborn Care
ENC: Essential Newborn Care
GAC: Global Affairs Canada
GBV: Gende-Based Violence
HBM: Hospital Based Mentors
HC: Health center
HCP: Health Care Provider
IPC: Inter-Professional Collaboration
LIC: Low Income Country
LMICs: Low-Middle-Income Countries
MCH: Maternal and Child Health
MDGs: Millennium Development Goals
MESH: Mentoring and Enhanced Supervision at Health Centers
MMR: Maternal Mortality Ratio
MNC: Maternal and Newborn Care
MNCH: Maternal,Newborn and Child Health
MNH: Maternal and Newborn Health
MoU: Memorandum of Understanding
PI: Principal Investigator
SDG: Sustainable Development Goals
TSAM: Training Support and Access Model
WHO: World Health Organization
